# Characterizing Hospital Workers' Willingness to Respond to a Radiological Event

**DOI:** 10.1371/journal.pone.0025327

**Published:** 2011-10-27

**Authors:** Ran D. Balicer, Christina L. Catlett, Daniel J. Barnett, Carol B. Thompson, Edbert B. Hsu, Melinda J. Morton, Natalie L. Semon, Christopher M. Watson, Howard S. Gwon, Jonathan M. Links

**Affiliations:** 1 Department of Epidemiology, Faculty of Health Sciences, Ben-Gurion University of the Negev, Beer-Sheva, Israel; 2 Clalit Health Services, Clalit Research Institute, Tel-Aviv, Israel; 3 Department of Emergency Medicine, Johns Hopkins University School of Medicine, Baltimore, Maryland, United States of America; 4 Johns Hopkins Office of Critical Event Preparedness and Response, Baltimore, Maryland, United States of America; 5 Johns Hopkins Preparedness and Emergency Response Research Center, Baltimore, Maryland, United States of America; 6 Department of Environmental Health Sciences, Johns Hopkins Bloomberg School of Public Health, Baltimore, Maryland, United States of America; 7 Johns Hopkins Public Health Preparedness Programs, Baltimore, Maryland, United States of America; 8 Department of Biostatistics, Johns Hopkins Bloomberg School of Public Health, Baltimore, Maryland, United States of America; 9 Department of Anesthesiology and Critical Care Medicine, Johns Hopkins University School of Medicine, Baltimore, Maryland, United States of America; 10 Department of Pediatrics, National Naval Medical Center, Bethesda, Maryland, United States of America; 11 Office of Emergency Management, Johns Hopkins Health System, Johns Hopkins University School of Medicine, Baltimore, Maryland, United States of America; Finnish Institute of Occupational Health, Finland

## Abstract

**Introduction:**

Terrorist use of a radiological dispersal device (RDD, or “dirty bomb”), which combines a conventional explosive device with radiological materials, is among the National Planning Scenarios of the United States government. Understanding employee willingness to respond is critical for planning experts. Previous research has demonstrated that perception of threat and efficacy is key in the assessing willingness to respond to a RDD event.

**Methods:**

An anonymous online survey was used to evaluate the willingness of hospital employees to respond to a RDD event. Agreement with a series of belief statements was assessed, following a methodology validated in previous work. The survey was available online to all 18,612 employees of the Johns Hopkins Hospital from January to March 2009.

**Results:**

Surveys were completed by 3426 employees (18.4%), whose demographic distribution was similar to overall hospital staff. 39% of hospital workers were not willing to respond to a RDD scenario if asked but not required to do so. Only 11% more were willing if required. Workers who were hesitant to agree to work additional hours when required were 20 times less likely to report during a RDD emergency. Respondents who perceived their peers as likely to report to work in a RDD emergency were 17 times more likely to respond during a RDD event if asked. Only 27.9% of the hospital employees with a perception of low efficacy declared willingness to respond to a severe RDD event. Perception of threat had little impact on willingness to respond among hospital workers.

**Conclusions:**

Radiological scenarios such as RDDs are among the most dreaded emergency events yet studied. Several attitudinal indicators can help to identify hospital employees unlikely to respond. These risk-perception modifiers must then be addressed through training to enable effective hospital response to a RDD event.

## Introduction

The number of terrorist bombings in the world has risen in the last 10 years [1], [2]. Examples such as the bombings in Bali (2002, 2005), Istanbul (2003), Madrid (2004), Egypt (2005), London (2005), and Mumbai (2006, 2009) have demonstrated the need for effective and rapid response in order to minimize casualties. A radiological dispersal device (RDD, or “dirty bomb”) combines a conventional explosive device with radiological materials. This type of device has the potential to generate greater effects on the targeted population than a conventional explosive device - due not only to radiological injury, but perhaps more importantly due to the profound economic and psychological impact of such an attack [3]–[5]. Although yet to be deployed successfully, the potential use of a RDD as a terrorist weapon is of significant concern to the United States government; deployment of a RDD is one of the fifteen Department of Homeland Security National Planning Scenarios [6]. Response to a dirty bomb detonation was exercised on the national level in TOPOFF 2, in which simulated use of a RDD in Seattle was one of the simultaneous modes of attacks used by hypothetical international terrorists [7].

In addition to psychological trauma to affected populations, RDD events may result in physical injuries and variable levels of radiation contamination. Severity of physical injuries depends on the nature of the explosive used, and extent of contamination is based on the degree of dispersal of the associated radiation. The physical health consequences from the RDD blast and its dispersed radiation would likely be limited to a maximum area of a few city blocks; further, the most significant contributor to injury and mortality from a RDD would be from the blast rather than the radiation [8]. Indeed, any victim close enough to receive an acute lethal radiation dose would likely have been killed by the blast itself [9]. For most people directly involved in a RDD scenario, it has been estimated that the exposure would carry a lifetime health risk comparable to that from smoking five packages of cigarettes or the accident risk of taking a hike [10]. Thus, from a public health perspective, a RDD is much more of a psychological weapon than a physical one [11].

Experts agree that while a dirty bomb is unlikely to sicken or kill many people from a public health perspective, it is very likely to cause fear and panic in the general population [12], [13] and is likely to result in disaster activation at local hospitals. The potential panic engendered by such an attack may overwhelm local health care facilities with individuals experiencing psychological rather than physical effects from the hazard itself [14]. The Centers for Disease Control and Prevention (CDC) estimates that 50–80% of victims of an explosive event arrive at medical facilities within the first 90 minutes [15] which illustrates the importance of hospitals being prepared to rely on their own staff for an initial rapid response to a radiological event, rather than relying heavily on external response assets. A growing body of emergency preparedness literature among a variety of care providers—including emergency medical technicians (EMTs) [16], local public health department workers [17], [18], and urban healthcare workers [19]–[22] —indicates that willingness to respond during a disaster is a scenario-specific phenomenon. Furthermore, research suggests that response willingness is multidimensional and influenced by a variety of risk perception modifiers peripheral to the actual event, such as perception of the importance of one's role in the agency's overall response [23] and concerns about personal and family safety [10]. Personal safety concerns may play an even larger role for health care responders in the dirty bomb scenario than in many other types of disaster scenarios, given factors such as the risk of exposure to the radiological contaminant, potential lack of adequate protective equipment, and the unusual nature of this type of attack [23]–[25].

The willingness of hospital-wide staff to respond to a RDD has not yet been assessed. One framework that has proved applicable in assessing root causes of willingness to respond to duty during emergencies has been Witte's Extended Parallel Process Model (EPPM). This framework allows for examining the interplay and influence of perceptions of “threat” and “efficacy” on adaptive or maladaptive behavior of healthcare workers in deciding whether to report to duty in the face of risk [26].

We have set out to assess the willingness of employees at a large, urban, tertiary-care medical center to respond during a RDD event. Accordingly, we aim to gauge whether a clinically significant proportion of the workforce may be unwilling to respond to duty during an RDD event, and whether specific personal characteristics and beliefs are independently associated with willingness to respond in this event. We further analyze the influences of perceived threat and perceived efficacy among hospital employees utilizing Witte's EPPM and attempt to identify factors potentially influencing willingness and ability to respond.

## Methods

### Study Setting

The survey was administered at the Johns Hopkins Hospital (JHH) in metropolitan Baltimore, Maryland. JHH is a Level 1 Trauma Center with 982 beds. It is the major teaching center for the Johns Hopkins School of Medicine and School of Nursing.

### Ethics Statement

Research ethics approval for the survey and its administration was received from The Johns Hopkins Medicine Institutional Review Board (JHM IRB) with a waiver of written consent. Study materials included an electronic disclosure describing the study and emphasizing voluntary participation; verbal consent was not requested or required by JHM IRB.

### Study tool

The survey tool, entitled “Disaster Preparedness and Emergency Response Survey”, was an anonymous online instrument (SurveyMonkey.com, Portland, OR) consisting of two main sections: a demographic section and an attitude/belief section that focused on hospital workers' attitudes and beliefs toward emergency response. The demographic and professional information included are listed in Table S1.

For the RDD scenario, a series of attitude and belief statements were presented for level of agreement along with two open-ended questions. Responses to the attitude and belief statements were based on a 9-point Likert scale with a response of ‘1’ indicating strong agreement with the statement, a response of ‘5’ indicating neutrality, and a response of ‘9’ indicating strong disagreement with the statement. Respondents could also indicate “don't know”. These attitudes and beliefs are detailed in the Results section. Two main contexts for willingness to respond (“WTR”) to a RDD were also assessed - WTR if asked but not required to respond (hereafter referred to as “WTR if asked”), and WTR if required, were presented using the 9-point Likert scale.

In accordance with the EPPM-based threat and efficacy methodology validated by multiple studies and explained in previous work [17], [18], [27], [28], levels of perceived threat and perceived efficacy (both with regards to the individual respondent) were determined, and four profiles were constructed.

### Study participants

All employees of the Johns Hopkins Hospital (N = 18,612) were designated as eligible for participation in the survey, which was conducted from January 2, 2009 to March 9, 2009. Study notification and requests for voluntary participation were distributed via department manager announcements, hospital-wide emails, posters, and informational plasma screens throughout the hospital.

### Statistical Analysis

Prior to analysis, responses to the attitude and belief statements were dichotomized into categories of ≤4 (‘positive response’) versus ≥5 (‘negative response’). One of the four EPPM profiles was assigned to each respondent using the low and high perceived threat and efficacy categories calculated as described in previous EPPM survey-based research [17], [18], [27], [28].

Distributions of demographic/professional factors and agreement with attitude/belief statements were obtained with respect to the two main WTR contexts noted above. Univariate logistic regression analyses were performed to determine key demographic factors most predictive of a positive response to the main WTR contexts. Multivariate logistic regression analyses, adjusting for the key demographic factors, were then performed to evaluate the attitude/belief statements, EPPM profiles, and training scenarios predictive of a positive response for each of the main WTR contexts. Missing and “don't know” responses were excluded from the analyses. All analyses were performed using STATA version 11.1 (STATA Corporation, 2010; College Station, TX).

## Results

Responses to the online survey were received from 3426 JHH employees. This sample constitutes 18.4% of JHH staff. An accurate estimate for response rate is difficult to assess, as it was not possible to ascertain what proportion of JHH staff have indeed been exposed to the email invitation to participate in the survey. Key characteristics of the respondents are detailed in Table S1; JHH staff data on age, gender and professional category show that this sample is representative of the overall JHH staff characteristics.

Among the respondents, 27.3% were male, and 72.7% were female; 16.5% were younger than 30 years, 47.5% were aged 30–49 years, and 36% were aged 50 and older. Thirty-four percent of the respondents were clinical staff, and 66% were non-clinical (the latter including food service/linens, IT, legal, executive officers, nursing administration, parking, pharmacy, safety, social workers, supply chain, telecommunications, etc). Of the 1170 clinical respondents, 42.7% were physicians, 49.2% were nurses, and 8.1% were “other” (physician extenders and medical/nursing students). The majority of respondents (60.7%) had worked in the hospital for 10 or fewer years.

Table S2 details the percent agreement with key attitudes and belief statements. Of note is the fact that 88% of the respondents considered a RDD event likely to be of severe health consequences, that only 41.9% felt they were knowledgeable about this threat, that only 35.8% felt they were able to address public questions on this threat, and only 31.9% were aware of their job-specific responsibilities in such an event. The average “don't know” response proportion was generally stable across strata (7–13%). One single stratum, the lowest education level, had a higher proportion of “don't know” responses (16%),

Overall willingness to respond to a RDD scenario was 61% if asked, and 72% if required. Table S1 shows that higher levels of WTR if asked (“unadjusted” for key demographic characteristics) were associated with older age [OR(95%CI) of 1.56 (1.06, 2.77) for ages 60+ as compared with ages <30]; and for males compared to females [OR(95%CI): 1.87 (1.53, 2.30)]. In addition, a significantly lower unadjusted likelihood of WTR if asked was evident for those having children and married [OR(95%CI): 0.69 (0.57, 0.83)] and single parents [OR(95%CI): 0.56 (0.41, 0.77)], compared to those having no children regardless of marital status; for those having pets [OR(95%CI): 0.79 (0.66, 0.95)] compared to those without pets; and for nurses and “other” hospital workers [OR(95%CI): 0.53 (0.38, 0.72) and 0.46 (0.36, 0.60), respectively] compared to physicians. Other variables, including type of department (emergency medicine, clinical and non-clinical) had no significant association with WTR if asked. Except for the association with age, children/marital status, elder family members, and hours working, the other associations held similarly for WTR if required.

In a multivariate analysis, four of the demographic and professional factors (gender, age, marital status/dependent children, and professional category) were found to be independently associated with both WTR if asked and WTR if required, and are used as adjustors in subsequent analyses.

After adjusting for these demographic factors, several attitude/belief statements had a significant association with WTR if asked (Table S2): perception that colleagues will report [OR(95%CI): 16.99 (13.06, 22.10)]; perceived high impact of one's response [OR(95%CI): 6.42 (5.14, 8.03)]; feeling psychologically prepared to perform one's role-specific responsibilities in the event [OR(95%CI): 8.08 (6.41, 10.18)]; perceived confidence one would be safe at work [OR(95%CI): 12.24 (9.21, 16.26)]; perceived confidence one could safely get to work [OR(95%CI): 8.58 (6.76, 10.90)]; perceived ability to perform one's duties [OR(95%CI): 9.27(7.35, 11.70)]; and perception that family is prepared to function in one's absence [OR(95%CI): 7.73 (6.18, 9.66)].

Associations of attitude/belief statements with the two WTR contexts were also evaluated to consider staff who would respond neither if asked nor if required (Table S3). Although significant associations remain, they were generally smaller for WTR if required and larger for WTR if asked than the associations in Table S2 which considered a person's response to each WTR context individually.

When questioned about potential modifiers of willingness to respond (conditional willingness to respond), WTR increased to 83.7% if daily preventive medications were made available (compared to WTR of 61% if asked). Clarifications of potential worker safety issues considerably reduced WTR rates, compared to WTR if asked: if personal protective equipment was *not* available for all staff (36.3%), and if a severe event was considered (50.7%). Only 27.9% of the hospital employees with a perception of low efficacy declared willingness to respond to a severe RDD event. 51% of surveyed staff indicated they are unlikely to respond to a RDD “regardless of its severity”.

In accordance with the EPPM, measures for threat and efficacy perception were calculated. When adjusting for the key demographic factors, higher perceived threat [OR(95%CI): 1.26 (1.03, 1.54)] and higher perceived efficacy [OR(95%CI): 6.89 (5.43, 8.75)] were associated with a higher WTR if asked (Table S2). When the threat and efficacy factors were combined into the four EPPM profiles, the High-Threat/High-Efficacy profile was associated with at least seven times higher odds of WTR if required and of WTR if asked, as compared to the odds for the reference low-threat/low-efficacy profile [OR(95%CI): 7.12 (4.91, 10.32), and 7.16 (5.12, 10.00), respectively]. The threat component of the profiles had no independent significant impact on WTR if required, and the high-threat/low-efficacy profile had no advantage over the low-threat/low-efficacy profile in either WTR if asked or WTR if required. This similarly applies to the high-efficacy comparison between threat levels.

Table 1 lists associations between self-reported willingness to respond to a RDD emergency and respondents' training and disaster experiences. Only 50% of the respondents had received some form of training, and less than 14% had undergone an RDD drill. Those respondents that had no RDD training were almost 1.5 times more likely not to be willing to respond to duty even if required, compared to those with at least some training. Participants that had both disaster management training and disaster experience were 6 times more likely to respond to a RDD event, adjusted for the four key demographic factors associated with WTR.

**Table 1:** Associations between self-reported willingness to respond (WTR) to a radiological dispersal device emergency and respondents' training and disaster experiences.

			WTR if required		WTR if asked but not required	
Training/Disaster Experience		%^a^	% Agree^b^	OR^c^ (95% CI)^d^	% Agree	OR (95% CI)
Any training	Some	50.6	75.5	Reference	64.5	Reference
	None	49.4	69.1	0.69 (0.57–0.84)	58.2	0.74 (0.62–0.891)
Tabletop exercise(s)	No	84.0	71.2	Reference	60.0	Reference
	Yes	16.0	78.1	1.38 (1.03–1.82)	68.7	1.40 (1.09–1.81)
Full-scale drills(s)/exercise(s)	No	85.9	71.3	Reference	60.3	Reference
	Yes	14.1	78.7	1.53 (1.13–2.07)	68.0	1.36 (1.03–1.78)
Academic coursework	No	85.5	71.0	Reference	59.3	Reference
	Yes	14.5	79.8	1.46 (1.08–1.97)	73.0	1.66 (1.25–2.20)
Face-to-face training(s)/lecture(s)/presentation(s)	No	80.0	70.5	Reference	59.2	Reference
	Yes	20.0	79.6	1.63 (1.25–2.18)	70.0	1.58 (1.25–2.00)
Online training module(s)	No	89.0	71.7	Reference	60.2	Reference
	Yes	20.0	75.0	1.26 (0.98–1.61)	66.1	1.40 (1.11–1.77)
Writing emergency/disaster management (EM) plans	No	91.9	71.5	Reference	60.2	Reference
	Yes	8.1	81.7	1.96 (1.31–2.95)	74.3	2.11 (1.48–3.03)
Real-life disaster experience	No	93.6	71.5	Reference	60.5	Reference
	Yes	6.4	84.9	2.02 (1.24–3.27)	74.5	1.64 (1.09–2.46)
Disaster experience or training	No	51.4	68.8	Reference	57.9	Reference
	Yes	48.6	75.9	1.50 (1.23–1.83)	64.9	1.39 (1.16–1.68)
No training or disaster experience		51.4	68.8	Reference	57.9	Reference
Disaster experience only		1.4	80.0	1.72 (0.69–4.30)	69.0	1.41 (0.62–3.21)
Any training only		42.2	74.6	1.42 (1.12–1.74)	63.5	1.34 (1.10–1.62)
Any training and disaster experience		5.0	86.2	2.62 (1.48–4.62)	75.9	2.03 (1.27–3.25)
No EM training or disaster experience		87.2	71.0	Reference	59.7	Reference
Disaster experience only		4.7	81.4	1.65 (0.98–2.76)	69.0	1.31 (0.85–2.05)
EM training only		6.4	78.0	1.70 (1.11–2.61)	70.4	1.86 (1.26–2.74)
EM training and disaster experience		1.7	94.6	6.19 (1.43–26.79)	89.2	4.70 (1.63–13.58)

a Percent of respondents included in category.

b Percent agreeing with WTR statement (positive response).

c OR is the odds ratio provided in the logistic regression which compares the odds between a positive WTR response and a negative WTR response with respect to the type of training compared to its Reference category, adjusted for key demographic characteristics: gender, age, children/marital status, and type of professional category.

d 95%CI is the 95% confidence interval for the odds ratio.

## Discussion

Our study suggests that during a RDD or “dirty bomb” event, a high proportion (39%) of the medical center staff may opt out from responding to duty, and that several attributes, most importantly willingness to work extra hours (associated with having dependent family members and pets at home) are very strongly associated with willingness to respond in such an emergency.

The use of a RDD or “dirty bomb” as a terrorist weapon is a concern, as reflected by its inclusion among the U.S. National Planning Scenarios. Psychological models suggest that risk perception is an interplay between affective (risk as feeling) and analytic (risk as analysis) processes [29]. According to these models, peripheral factors independent of the actual risk have a major effect on the perceived dread of an event. Factors that render a perceived risk as more dreadful include events that are involuntary, manmade, exotic, catastrophic, and with potential to affect the next generation with little or no individual control. Virtually each and every one of these risk perception modifiers is present in a RDD scenario, rendering this event to be highly dreadful to some, well beyond the actual (analytic) risks it bears.

During critical events, healthcare workers are expected to work additional hours under significant stress, potentially at risk of personal safety. When faced with the need to respond to duty during a terrorist event, health professionals are subject to the psychological impact of dread and outrage, caused by the perception-modifying characteristics of a RDD event. This may explain, at least in part, why such a large proportion of hospital workers, almost 39%, report they would not be willing to report to duty if asked during a RDD event. When further probed if they would respond to a RDD event “regardless of severity”, almost half (51%) of surveyed staff indicated they are unlikely to do so. This is a very high proportion when considering the first receiver role of these personnel, ostensibly accustomed to responding to emergencies and disasters.

These outcomes are in accordance with the limited but expanding evidence-based literature on the perceptions of the hospital-based workforce toward their emergency response duties in a post-9/11 world. In two surveys performed in 2005 of NYC healthcare workers (n = 6,428), and hospital employees in 5 states (n = 1711), workers were far more willing to respond to natural disasters than to a radiological event or an infectious disease outbreak [19]. Yet the results of our study regarding WTR in a RDD event are markedly worse than the 28% who were unwilling to respond to a pandemic influenza event, measured in the same setting and population (just prior to the 2009 H1N1 pandemic) [27], [28].

Having a workforce that is willing to respond is a critical component of mitigating the effects of any disaster, and our study results are a clear call for action. While work is being done by disaster planners to improve “readiness” or “ability” to respond during disasters, such as encouraging personal preparedness planning, more needs to be done to address beliefs and attitudes that may hinder “willingness” to respond. It is thus critically important for us to understand why some healthcare workers are unwilling to perform their duties during a radiological emergency in order to implement changes in disaster training, education and messaging.

Survey responses suggest that more attention is needed to address healthcare workers' basic knowledge level with regard to radiation events. In fact, 58% of respondents disagreed with the statement “I am knowledgeable about the potential medical impacts of a dirty bomb emergency.” Two thirds of the staff surveyed did not feel educated enough to address public questions, and less than one third of the staff knew their role-specific responsibilities. Indeed, in a recent study of 668 emergency nurses in New York, the existing knowledge in regards to radiological emergencies was determined to be poor [19]. In that study, knowledge level and clinical ability had a positive association with nurses' level of willingness to respond to a radiological terrorism event.

Quantitative results from our hospital-based study also echo a qualitative study that assessed the views and perspectives of emergency department clinicians in regard to radiologic terrorism [30]. Researchers found through a series of ten focus groups that study participants clearly and consistently felt that their facilities were not adequately prepared for such an event, due to inadequacy of response protocols, potential for staffing shortages, and concerns about contamination and self-protection. When considering the fear of potential staffing shortages indeed, in our study, staff who felt that their peers are unlikely to respond to duty, were 17 times more likely to refrain from reporting to duty themselves in our study. This finding lends us a potentially powerful tool to impact willingness to respond, by targeted education campaigns to change subjective norms regarding response to such an emergency.

One construct that was strongly and independently associated with WTR was belief that the workplace will be safe. Perception of personal safety was identified as a primary determinant of willingness to respond in a radiological disaster in other previous work as well [23]. This concern about personal protection is not unique among responders to the potential scenario of radiological terrorism events; in one study the question of “Will the hospital protect me?” was the most important factor in determining the workers willingness to respond [31]. First responders must be educated as to the minimal risk of contamination from radiologic materials in such an attack if universal precautions are used [32], as well as in specific strategies of mitigating and minimizing personal risk in such events. Thus, it is not surprising that requiring the staff to report will not be enough to address the worker shortage. Unwillingness of hospital staff to respond to a RDD event remained as high as 27.6% if the workers were required (and not just asked) to report to duty. Thus, even the potential threat of loss of compensation or job is of limited influence, as still over one of every four employees from a large urban tertiary care hospital indicated they would not report for work even if required – at a time they would be most needed in their respective work roles.

Of all attitudes and beliefs, the attitude statements most strongly associated with high WTR was willingness to work extra hours if required. Those able and willing to work extra hours were 20 times more likely to be willing to respond during this event, after controlling for demographic factors. These results may be interpreted in view of the strong association identified between lower ‘if asked’ WTR and having dependents at home – either elderly (OR = 0.81), children (OR = 0.69) or even pets (OR = 0.79). Single parents with children had the lowest estimated likelihood to respond to this event (OR = 0.56). One reasonable explanation may be that some of the hesitance to report to duty among those unable to work long hours is associated with the need to continuously take care of dependents during such an event. Indeed, 88% of those unwilling to work extra hours had a family member or a pet dependent solely on them.

Witte's EPPM offers a framework for examining the interplay and influence of perceptions of “threat” and “efficacy” on adaptive or maladaptive behavior in the face of risk [26]. It has shown its utility in previous work assessing WTR in pandemic influenza and other catastrophic event scenarios [18], [27], [28]. Our study is the first to analyze hospital employees' perceived threat, efficacy, and WTR during a RDD event through the lens of the EPPM. This model potentially allows us to see how hospital workers' individual degrees of perceived threat (“concern”) and perceived efficacy (“confidence”) influence their willingness to respond to this type of event. In accordance with EPPM theory, our survey results show that those who have a perception of high threat and high efficacy – i.e., those who fit a “concerned and confident” profile in the EPPM framework—had a high rate of declared self-reported willingness to respond (if required) to a dirty bomb event, which was about seven times [OR(95%CI): 7.16 (5.12–10)] higher than those fitting a “low threat/low efficacy” profile.

In contrast with the classic EPPM theory, perception of threat had little impact on willingness to respond among hospital workers in our study (Table S2). This could either imply that the perception of threat in motivating response behavior in hospital employees is not as important as the perception of one's efficacy in response, or that our threat assessment questions assessed the ‘analytic’ aspect of RDD risk perception, and could not assess the ‘affective’ effect of the additional dread associated with this event, which is the effect that may impact WTR more significantly. One other potential explanation is that the level of dread from such a scenario is such that only minor variability exists between individuals, in a level that bears little impact on decision making.

Our survey indicates that hospital employees are receptive to more training in response to a radiation disaster. In fact, 87% of respondents agreed that the hospital should provide pre-event preparation and training for dirty bomb emergencies. Only 50% of the respondents had received some form of training. Those who had were almost 1.5 times more likely to be willing to respond to duty even if not required than those with no training. Participants that had both disaster management training and disaster experience were 6 times more likely to respond to a RDD event, adjusted for four demographic characteristics associated with WTR. Most preparedness training for hospital workers presents factual information on threats and response roles. Classroom and web-based educational interventions are generally awareness-level courses that focus on knowledge objectives, such as the nature of disasters or terrorist events, medical effects, treatment modalities, personal protective equipment, and immuno- or chemo-prophylaxis if available [33], [34]. Some practicum courses have added skills objectives through hands-on workshops [35]. Additional strategies to mitigate such concerns include the application of internet-based learning courses on aspects of radiation emergency management and hazard mitigation [36], as well as conferences or medical symposia to educate medical professionals on the aspects of patient management in radiation injury scenarios [37], and extensive preparatory planning [38].

Our study had several key limitations that must be considered when interpreting its results. We have used an online survey, and thus some members of hospital staff may have had unequal opportunity to respond to it, despite the availability of computers all around the hospital accessible to all employees. However, the large number of respondents, representative of the entire hospital staff, may point to its internal validity. This study was limited to one institution, thus limiting its external validity. Despite this, the study allows us to consider these results a high-level estimate for other non-tertiary centers around the country. It is theoretically possible that an individual could have responded more than once to the survey, although the authors believe this is very unlikely given the length of time required to fill out the survey. Finally, there is always a concern about the difference between the declared responses and actual conduct when facing the actual risk. Again, one can assume that these results are thus conservative, and actual willingness to respond may be lower but is unlikely to be higher in a real-life event.

### Conclusions

Our study demonstrates a significant gap that exists in hospital preparedness for a ‘dirty bomb’ radiological terrorism event, with nearly 40% of the workers unwilling to respond to duty during the event. The subjective norm (perceived willingness of peers to respond), personal safety issues, and perceived efficacy in one's role in response were found to be important parameters associated with willingness to respond, while the level of perceived threat had only minor impact. These data, in view of the considerable gaps in perceived knowledge and training identified, lay the evidence needed to guide future preparedness and curriculum planning for hospital employees and to identify critical incentives for the hospital workforce response during this type of disaster.

## Supporting Information

Table S1 **Associations between demographic characteristics and self-reported WTR to a radiological dispersal device emergency.**
(DOC)

Table S2 **Associations between attitudes/beliefs and self-reported WTR to a radiological dispersal device emergency.**
(DOCX)

Table S3 **Associations between attitudes/beliefs and self-reported WTR to a radiological dispersal device emergency compared to those not willing to respond.**
(DOC)
