# Supplementary material for: Characterizing Hospital Workers' Willingness to Respond to a Radiological Event
Source: PLoS One. 2011 Oct 27;6(10):e25327. doi: 10.1371/journal.pone.0025327 (PMC3203116; doi:10.1371/journal.pone.0025327)
Supplement: Table S1 — Associations between demographic characteristics and self-reported WTR to a radiological dispersal device emergency. (DOC) [file pone.0025327.s001.doc]

**Table S1. Associations between respondents’ demographic characteristics and self-reported willingness to respond (WTR) to a radiological dispersal device emergency**

|  |  |  | **WTR if required** | | | **WTR if asked but not required** | | | |
| --- | --- | --- | --- | --- | --- | --- | --- | --- | --- |
|  |  | **%^a^** | **% Agree^b^** | **OR^c^** | **(95%CI)^d^** | **% Agree** | **OR** | | **(95%CI)** |
| **Overall^e^** |  |  | 72.4 |  |  | 61.4 |  | |  |
| **By respondent characteristics** | |  |  |  |  |  |  | |  |
| Gender | Female | 73 | 69.4 | Reference | | 57.1 | Reference | | |
|  | Male | 27 | 79.6 | 1.72 | (1.37 - 2.16) | 71.4 | 1.87 | (1.53 - 2.30) | |
| Age (years) | <30 | 17 | 69.9 | Reference | | 59.6 | Reference | | |
|  | 30-39 | 22 | 68.2 | 0.93 | (0.67 - 1.27) | 57.2 | 0.91 | (0.67 - 1.22) | |
|  | 40-49 | 26 | 72.3 | 1.13 | (0.82 - 1.54) | 59.5 | 0.99 | (0.74 - 1.33) | |
|  | 50-59 | 27 | 75.0 | 1.29 | (0.95 - 1.76) | 64.4 | 1.23 | (0.92 - 1.63) | |
|  | 60+ | 9 | 77.3 | 1.47 | (0.97 - 2.21) | 69.6 | 1.55 | (1.06 - 2.27) | |
| Duration at JHH^f^  (years) | <1 | 11 | 75.5 | Reference | | 65.2 | Reference | | |
|  | 1-5 | 33 | 72.4 | 0.85 | (0.59 - 1.23) | 61.8 | 0.87 | (0.62 - 1.21) | |
|  | 6-10 | 17 | 71.0 | 0.79 | (0.53 - 1.18) | 58.8 | 0.76 | (0.53 - 1.10) | |
|  | >10 | 39 | 72.2 | 0.84 | (0.59 - 1.20) | 61.3 | 0.85 | (0.61 - 1.17) | |
| Hours/week working  at JHH | <10 | 4 | 71.8 | Reference | | 66.7 | Reference | | |
|  | 10-19 | 1 | 80.7 | 1.64 | (0.60 - 4.50) | 58.6 | 0.71 | (0.30 - 1.69) | |
|  | 20-29 | 3 | 63.9 | 0.70 | (0.34 - 1.41) | 52.5 | 0.55 | (0.28 - 1.10) | |
|  | 30-39 | 10 | 70.1 | 0.92 | (0.53 - 1.61) | 55.9 | 0.63 | (0.37 - 1.08) | |
|  | 40-49 | 66 | 69.7 | 0.91 | (0.56 - 1.47) | 58.0 | 0.69 | (0.43 - 1.10) | |
|  | 50+ | 16 | 83.1 | 1.94 | (1.13 - 3.33) | 75.8 | 1.56 | (0.94 - 2.60) | |
| Worked in JHH role  (years) | <1 | 13 | 74.1 | Reference | | 66.5 | Reference | | |
|  | 1-5 | 37 | 70.8 | 0.85 | (0.61 - 1.18) | 60.6 | 0.77 | (0.57 - 1.05) | |
|  | 6-10 | 17 | 74.9 | 1.05 | (0.72 - 1.52) | 58.8 | 0.72 | (0.51 - 1.01) | |
|  | >10 | 34 | 72.5 | 0.92 | (0.66 - 1.28) | 61.7 | 0.81 | (0.60 - 1.10) | |
| Highest education  level completed | Professional | 19 | 81.8 | Reference | | 72.4 | Reference | | |
|  | MS | 20 | 72.0 | 0.57 | (0.42 - 0.79) | 62.4 | 0.63 | (0.48 - 0.84) | |
|  | Bachelors | 36 | 72.1 | 0.58 | (0.43 - 0.77) | 60.1 | 0.57 | (0.44 - 0.74) | |
|  | HS/GED | 24 | 63.4 | 0.39 | (0.28 - 0.53) | 50.9 | 0.39 | (0.30 - 0.52) | |
| Rely on public  transportation | No | 82 | 73.0 | Reference | | 61.4 | Reference | | |
|  | Yes | 18 | 71.0 | 0.91 | (0.70 - 1.18) | 63.2 | 1.08 | (0.84 - 1.38) | |
| Have elder family members who rely on  you for care | No | 78 | 74.2 | Reference | | 62.6 | Reference | | |
|  | Yes | 22 | 66.5 | 0.69 | (0.55 - 0.86) | 57.5 | 0.81 | (0.65 - 1.00) | |
| Children/marital  status | No children | 54 | 74.4 | Reference | | 66.0 | Reference | | |
|  | Children/Single | 10 | 65.0 | 0.64 | (0.46 - 0.90) | 52.2 | 0.56 | (0.41 - 0.77) | |
|  | Children/Married | 36 | 71.4 | 0.86 | (0.70 - 1.06) | 57.2 | 0.69 | (0.57 - 0.83) | |
| Have pets who rely  solely on you | No | 44 | 74.8 | Reference | | 64.0 | Reference | | |
|  | Yes | 56 | 69.6 | 0.77 | (0.64 - 0.94) | 58.4 | 0.79 | (0.66 - 0.95) | |
| Type of profession | MD | 14 | 83.6 | Reference | | 74.3 | Reference | | |
|  | RN | 17 | 72.3 | 0.51 | (0.36 - 0.73) | 60.3 | 0.53 | (0.38 - 0.72) | |
|  | Other professional | 3 | 81.3 | 0.85 | (0.43 - 1.69) | 79.4 | 1.33 | (0.69 - 2.56) | |
|  | Other (non-prof) | 66 | 68.7 | 0.43 | (0.32 - 0.58) | 57.2 | 0.46 | (0.36 - 0.60) | |
| Department type | Emergency medicine | 4 | 79.2 | Reference | | 69.2 | Reference | | |
|  | Clinical | 72 | 72.7 | 0.70 | (0.42 - 1.16) | 61.5 | 0.71 | (0.45 - 1.12) | |
|  | Non-clinical | 24 | 70.6 | 0.63 | (0.37 - 1.07) | 59.8 | 0.66 | (0.41 - 1.06) | |

^a^ Percent of respondents in category within characteristic

^b^ Percent agreeing with WTR statement (positive response)

^c^ OR is the odds ratio provided in the logistic regression which compares the odds between a positive WTR response and a negative WTR response with respect to a particular characteristic category compared to its reference category, unadjusted for other demographic characteristics.

^d^ 95%CI is the 95% confidence interval for the odds ratio.

^e^ Percent covers all respondents.

^f^ Johns Hopkins Hospital (JHH)
