# Supplementary material for: Characterizing Hospital Workers' Willingness to Respond to a Radiological Event
Source: PLoS One. 2011 Oct 27;6(10):e25327. doi: 10.1371/journal.pone.0025327 (PMC3203116; doi:10.1371/journal.pone.0025327)
Supplement: Table S2 — Associations between attitudes/beliefs and self-reported WTR to a radiological dispersal device emergency. (DOCX) [file pone.0025327.s002.docx]

## Table S2. Associations between attitudes/beliefs and self-reported willingness to respond (WTR) to a radiological dispersal device emergency

|  |  | **WTR if required** | **WTR if asked but not required** |
| --- | --- | --- | --- |
| **Attitudes and beliefs** | **% Agree^a^** | **OR^b^ (95%CI)** | **OR (95%CI)** |
| Perceived likelihood of occurrence in this region | 38.1 | 1.05 | 1.12 |
|  |  | (0.84 - 1.30) | (0.91 - 1.37) |
| Perceived severe health consequences likely | 88.0 | 3.12 | 1.98 |
|  |  | (2.34 - 4.16) | (1.48 - 2.64) |
| Perceived likelihood of being asked to report to duty | 68.7 | 7.20 | 5.34 |
|  |  | (5.67 - 9.14) | (4.26 - 6.68) |
| Perceived likelihood that colleagues will report | 51.2 | 12.92 | 16.99 |
|  |  | (9.66 - 17.28) | (13.06 - 22.10) |
| Perceived knowledge about the medical impact | 41.9 | 2.67 | 2.94 |
|  |  | (2.12 - 3.36) | (2.38 - 3.63) |
| Perceived awareness of role-specific responsibilities | 31.9 | 3.55 | 4.02 |
|  |  | (2.67 - 4.71) | (3.12 - 5.19) |
| Perceived skills for role-specific responsibilities | 51.1 | 6.44 | 5.68 |
|  |  | (4.99 - 8.31) | (4.54 - 7.11) |
| Psychologically prepared | 50.3 | 8.96 | 8.08 |
|  |  | (6.84 - 11.73) | (6.41 - 10.18) |
| Perceived ability to safely get to work | 47.1 | 10.35 | 8.58 |
|  |  | (7.75 - 13.81) | (6.76 - 10.90) |
| Confidence in personal safety at work | 39.1 | 9.63 | 12.24 |
|  |  | (6.98 - 13.29) | (9.21 - 16.26) |
| Perceived ability to perform duties (Self Efficacy) | 54.5 | 10.26 | 9.27 |
|  |  | (7.86 - 13.40) | (7.35 - 11.70) |
| Perceived that family is prepared to function in absence | 49.2 | 8.47 | 7.73 |
|  |  | (6.54 - 10.97) | (6.18 - 9.66) |
| Self-reported willingness to perform duties if additional hours are required | 66.6 | 20.25 | 19.74 |
|  |  | (15.51 - 26.44) | (15.34 - 25.41) |
| Hospital's perceived ability to provide timely information | 63.5 | 4.22 | 4.21 |
|  |  | (3.38 - 5.26) | (3.41 - 5.19) |
| Perceived ability to address public questions | 35.8 | 5.13 | 5.57 |
|  |  | (3.88 - 6.79) | (4.35 - 7.13) |
| Perceived importance of one's role in the agency's overall response | 52.4 | 5.45 | 4.78 |
|  |  | (4.28 - 6.94) | (3.84 - 5.95) |
| Perceived need for pre-event preparation and training | 86.5 | 4.60 | 3.81 |
|  |  | (3.49 - 6.06) | (2.87 - 5.04) |
| Perceived need for during/post-event psychological support | 66.8 | 1.74 | 1.45 |
|  |  | (1.41 - 2.16) | (1.18 - 1.77) |
| Perceived high impact of one's response (Response Efficacy) | 62.6 | 7.55 | 6.42 |
|  |  | (5.93 - 9.60) | (5.14 - 8.03) |
| **EPPM**^c^ |  |  |  |
| EPPM - Low Threat | 51.9 | Reference | Reference |
|  |  |  |  |
| EPPM - High Threat | 48.1 | 1.2 | 1.26 |
|  |  | (0.97 - 1.49) | (1.03 - 1.54) |
| EPPM - Low Efficacy | 52.2 | Reference | Reference |
|  |  |  |  |
| EPPM - High Efficacy | 47.8 | 7.72 | 6.89 |
|  |  | (5.84 - 10.20) | (5.43 - 8.75) |
| EPPM - Low Threat/Low Efficacy | 31.7 | Reference | Reference |
|  |  |  |  |
| EPPM - Low Threat/High Efficacy | 21.4 | 12.90 | 8.06 |
|  |  | (7.80 - 21.34) | (5.59 - 11.61) |
| EPPM - High Threat/Low Efficacy | 21.4 | 1.21 | 1.09 |
|  |  | (0.91 - 1.63) | (0.82 - 1.45) |
| EPPM - High Threat/High Efficacy | 25.5 | 7.12 | 7.16 |
|  |  | (4.91 - 10.32) | (5.12 - 10.00) |

^a^ Percent agreeing with WTR statement (positive response)

^b^ OR is the odds ratio provided in the logistic regression which compares the odds between a positive WTR response and a negative WTR response with respect to the positive statement response compared to the negative statement response, adjusted for key demographic characteristics: gender, age, children/marital status, and professional category.

^c^ 95%CI is the 95% confidence interval for the odds ratio.

^d^ Extended Parallel Process Model
