# Supplementary material for: Characterizing Hospital Workers' Willingness to Respond to a Radiological Event
Source: PLoS One. 2011 Oct 27;6(10):e25327. doi: 10.1371/journal.pone.0025327 (PMC3203116; doi:10.1371/journal.pone.0025327)
Supplement: Table S3 — Associations between attitudes/beliefs and self-reported WTR to a radiological dispersal device emergency compared to those not willing to respond. (DOC) [file pone.0025327.s003.doc]

**Table S3. Associations between attitudes/beliefs and self-reported willingness to respond (WTR) to a radiological dispersal device emergency, as compared to those who are not willing to respond either if asked but not required or if required**

|  |  | **WTR if required^a^** | **WTR if asked but not required^b^** |
| --- | --- | --- | --- |
|  | **% Agree^c^** | **OR^d^ (95%CI)^e^** | **OR (95%CI)** |
| **Attitudes and beliefs** |  |  |  |
| Perceived likelihood of occurrence in this region | 38.1 | 1.13 | 1.12 |
|  |  | (0.79, 1.61) | (0.88, 1.42) |
| Perceived severe health consequences likely | 88.0 | 5.60 | 3.04 |
|  |  | (2.95, 10.64) | (2.22, 4.16) |
| Perceived likelihood of being asked to report to duty | 68.7 | 4.37 | 8.73 |
|  |  | (2.98, 6.42) | (6.68, 11.41) |
| Perceived likelihood that colleagues will report | 51.2 | 3.82 | 30.49 |
|  |  | (2.38, 6.14) | (20.94, 44.40) |
| Perceived knowledge about the public health impact | 41.9 | 1.49 | 3.56 |
|  |  | (1.00, 2.19) | (2.75, 4.61) |
| Perceived awareness of role-specific responsibilities | 31.9 | 1.37 | 4.6 |
|  |  | (0.86, 2.18) | (3.37, 6.30) |
| Perceived skills for role-specific responsibilities | 51.1 | 2.6 | 8.27 |
|  |  | (1.77, 3.82) | (6.21, 11)) |
| Psychologically prepared | 50.3 | 2.74 | 12.02 |
|  |  | (1.83, 4.09) | (8.92, 16.19) |
| Perceived ability to safely get to work | 47.1 | 4.11 | 14.43 |
|  |  | (2.70, 6.23) | (10.49, 19.95) |
| Confidence in personal safety at work | 39.1 | 2.36 | 15.96 |
|  |  | (1.42, 3.94) | (14.01, 23.13) |
| Perceived ability to perform duties (Self Efficacy) | 54.5 | 3.4 | 14.05 |
|  |  | (2.30, 5.02) | (10.43, 18.92) |
| Perceived that family is prepared to function in absence | 49.2 | 3.62 | 12.18 |
|  |  | (2.47, 5.33) | (9.09, 16.30) |
| Self-reported willingness to perform duties if additional hours are required | 66.6 | 6 | 39.29 |
|  |  | (4.10, 8.80) | (28.53, 54.13) |
| Hospital's perceived ability to provide timely information | 63.5 | 2.17 | 5.29 |
|  |  | (1.54, 3.07) | (4.13, 6.77) |
| Perceived ability to address public questions | 35.8 | 2.2 | 7.75 |
|  |  | (1.39, 3.48) | (5.61, 10.70) |
| Perceived importance of one's role in the agency's overall response | 52.4 | 2.51 | 6.97 |
|  |  | (1.71, 3.67) | (5.30, 9.16) |
| Perceived need for pre-event preparation and training | 86.5 | 2.51 | 5.47 |
|  |  | (1.63, 3.86) | (4.02, 7.43) |
| Perceived need for during/post-event psychological support | 66.8 | 1.94 | 1.9 |
|  |  | (1.35, 2.78) | (1.50, 2.40) |
| Perceived high impact of one's response (Response Efficacy) | 62.6 | 3.56 | 10.56 |
|  |  | (2.47, 5.12) | (8.08, 13.87) |
| **EPPM^f^** |  |  |  |
| EPPM - Low Threat | 51.9 | Reference | Reference |
|  |  |  |  |
| EPPM - High Threat | 48.1 | 0.8 | 0.72 |
|  |  | (0.56, 1.14) | (0.57, 0.91) |
| EPPM - Low Efficacy | 52.2 | Reference | Reference |
|  |  |  |  |
| EPPM - High Efficacy | 47.8 | 0.35 | 0.09 |
|  |  | (0.23, 0.53) | (0.07, 0.13) |
| EPPM - Low Threat/Low Efficacy | 31.7 | Reference | Reference |
|  |  |  |  |
| EPPM - Low Threat/High Efficacy | 21.4 | 4.76 | 16.52 |
|  |  | (2.42, 9.38) | (9.64, 28.30) |
| EPPM - HIgh Threat/Low Efficacy | 21.4 | 1.34 | 1.20 |
|  |  | (0.88, 2.06) | (0.87, 1.65) |
| EPPM - High Threat/HIgh Efficacy | 25.5 | 0.29 | 10.49 |
|  |  | (0.17, 0.51) | (6.88, 16.00) |

^a^ Comparison of respondents who are only willing to respond if required to respondents not willing to respond either if required or if asked but not required.

^b^ Comparison of respondents who are only willing to respond if asked but not required to respondents not willing to respond either if required or if asked but not required.

^c^ Percent agreeing with WTR statement (positive response)

^d^ OR is the odds ratio provided in the logistic regression which compares the odds between a positive WTR response and a negative WTR response with respect to the positive statement response compared to the negative statement response, adjusted for key demographic characteristics: gender, age, children/marital status, and professional category.

^e^ 95%CI is the 95% confidence interval for the odds ratio.

^f^ Extended Parallel Process Model
